# Interferon-Associated Transcriptional Responses Are Preserved in Human Asthmatic Airway Epithelial Cells During Viral Infection

**DOI:** 10.3390/ijms27146113

**Published:** 2026-07-08

**Authors:** Hamad H. Alanazi

**Affiliations:** Department of Clinical Laboratory Sciences, College of Applied Medical Sciences, Jouf University, Al-Qurayyat 77442, Saudi Arabia; hhalanazi@ju.edu.sa

**Keywords:** asthma, allergens, viral infections, interferons, airway epithelial cells, RNA viruses, asthma exacerbation, type 2 inflammation

## Abstract

Viral infections are the main cause of asthma exacerbation, particularly in children. Impaired interferon induction by the airway epithelium has been linked to increased asthma exacerbation during viral infections. Several studies have suggested that epithelial cells in asthmatic mice induce low levels of type I and type III interferons, which leads to increased viral load. However, emerging evidence suggests that epithelial cells from asthmatic individuals induce delayed interferon responses. This study aimed to assess the ability of asthmatic and healthy epithelial cells to mount interferon-associated responses after exposure to viral stimuli. Variations in gene expression associated with interferon response were analyzed using datasets obtained from the Gene Expression Omnibus (GEO) database. Airway epithelial cells derived from healthy and asthmatic individuals were infected with RNA virus and then subjected to microarray or RNA sequencing. Lung tissues obtained from the animal models (mice and rats) were analyzed using RNA sequencing. Further data analysis was performed using integrated differential expression and pathway analysis (iDEP). Viral infection of airway epithelial cells derived from healthy and asthmatic subjects induces strong expression of interferon- and interferon-related genes. Interferon-stimulated genes (ISGs) were robustly induced in both asthmatic and healthy human epithelial cells after viral infection. However, the induction of virus-induced interferon-related responses was significantly lower in the lung tissues of animals with pre-allergic inflammation. Although previous studies have reported that the antiviral-interferon response is impaired or diminished in asthmatic individuals, our findings suggest that interferon-associated transcriptional responses are preserved in the airway epithelial cells of asthmatics during viral infection. This suggests that asthmatic epithelial cells induce an antiviral immune response, characterized by the induction of interferon-associated genes necessary for viral removal. Future studies should investigate the mechanisms underlying virus-induced asthma exacerbation.

## 1. Introduction

Asthma is a chronic disease characterized by airway hyper-responsiveness, mucus overproduction, wheezing, and difficulty breathing. Approximately 300 million people worldwide are affected by asthma [1]. Several factors affect disease severity, including constant exposure to allergens, genetic susceptibility, and smoking. However, respiratory viral infections caused by DNA or RNA viruses represent a major trigger for asthma exacerbation in patients of all ages [2,3]. Respiratory tract infections (RTIs) are the most common infections worldwide [4]. The immune system protects the respiratory tract in several ways, including the production of mucus and antimicrobial components, such as defensins [5]. The airway epithelium is the first cellular layer that interacts with invading pathogens or allergens. During viral infections, immune receptors present in epithelial cells, such as Toll-like receptor 3 (TLR3) and retinoic acid-inducible gene I (RIG-I), sense the presence of viral double-stranded RNA (dsRNA) and initiate a signaling cascade essential for virus removal. These signaling cascades result in the release of type I and III interferons, which interfere with viral replication and stimulate immune responses to eliminate the virus. Allergens such as house dust mites (HDM) damage epithelial cells, causing the release of alarmins such as interleukin-25 (IL-25), IL-33, and thymic stromal lymphopoietin (TSLP), which lead to the activation of group 2 innate lymphoid cells 2 (ILC2) and initiation of type 2 lung inflammation [6,7,8,9].

Epithelial cells play an important role in shaping the subsequent immune response by releasing cytokines such as IL-1β and interferon-β (IFN-β) [10,11]. Binding of type I interferons (IFN-α and IFN-β) and type III interferons (IFN-λ) to the interferon-α/β receptor (IFNAR) and interferon lambda receptor 1 (IFNLR1) on other neighboring cells induces the activation of the Janus-activated kinase-signal transducer and activator of transcription (JAK/STAT) signaling pathway [12]. Activation of the JAK/STAT pathway leads to the induction of several ISGs [13]. For example, *OAS* (2′-5′ oligoadenylate synthetase) encodes an antiviral protein that activates RNase L to degrade viral RNA and block viral replication [14]. Additionally, ISG-encoded proteins, including myxovirus resistance (Mx) proteins and interferon-inducible transmembrane proteins (IFITMs), inhibit viral replication of a number of respiratory viruses [15,16]. Induction of interferon-related immune responses by epithelial cells is important, especially against viruses, such as influenza A virus (IAV) and rhinoviruses (RVs), since they are major triggers for asthma exacerbations [17].

Several studies have reported that the interferon response is either impaired or deficient in epithelial cells of asthmatic individuals [18,19]. However, emerging evidence suggests that interferon response is preserved or delayed in asthmatic airway epithelial cells [20,21,22,23]. Nonetheless, conflicting evidence has emerged regarding the ability of epithelial cells to induce an antiviral interferon response in the presence of inflammatory stress, as observed in asthma [22,24]. Understanding the mechanisms that affect interferon preservation in the airway epithelium is important for enhancing antiviral immunity and reducing asthma exacerbations during viral infections.

Recently, publicly available datasets have become a useful approach for identifying gene expression alterations associated with disease conditions such as asthma. In this study, we investigated several microarray and RNA sequencing (RNA-seq) datasets obtained from the Gene Expression Omnibus (GEO) database (http://www.ncbi.nlm.nih.gov/geo (accessed on 3 November 2025)). The analyzed datasets were derived from human samples and different animal models of asthma. To investigate interferon-related transcriptional responses during viral infection, we identified several differentially expressed genes (DEGs) in the lung tissues of animal models and airway epithelial cells derived from asthmatic and healthy individuals. DEGs were analyzed at baseline and after viral infection with different RNA viruses.

The preservation of the interferon immune response in the asthmatic airway epithelium is essential for appropriate antiviral defense [25,26]. However, whether asthmatic airway epithelium is capable of inducing an interferon response during viral infection is poorly understood. This study aimed to investigate the preservation of interferon-associated transcriptional responses in asthma using transcriptomic datasets.

## 2. Results

**Figure 1:**
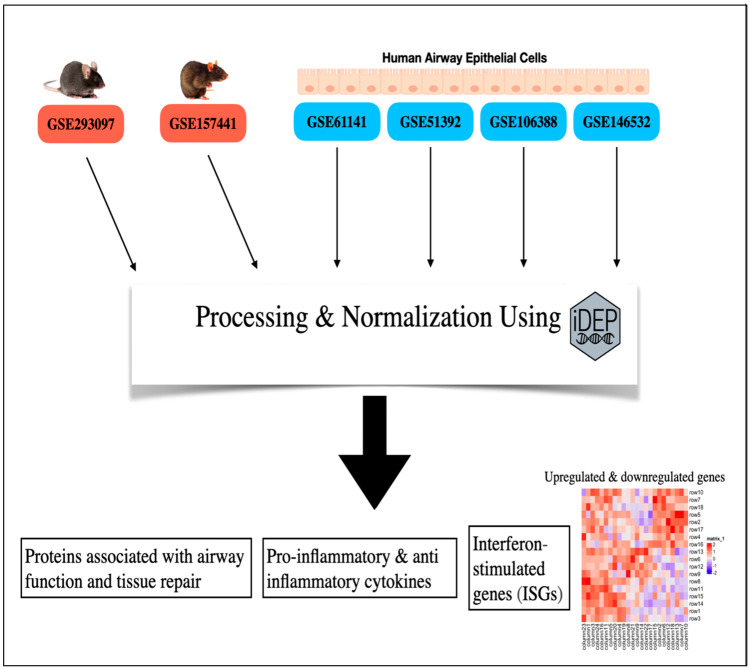
Flowchart showing dataset selection and analysis.

**Figure 2:**
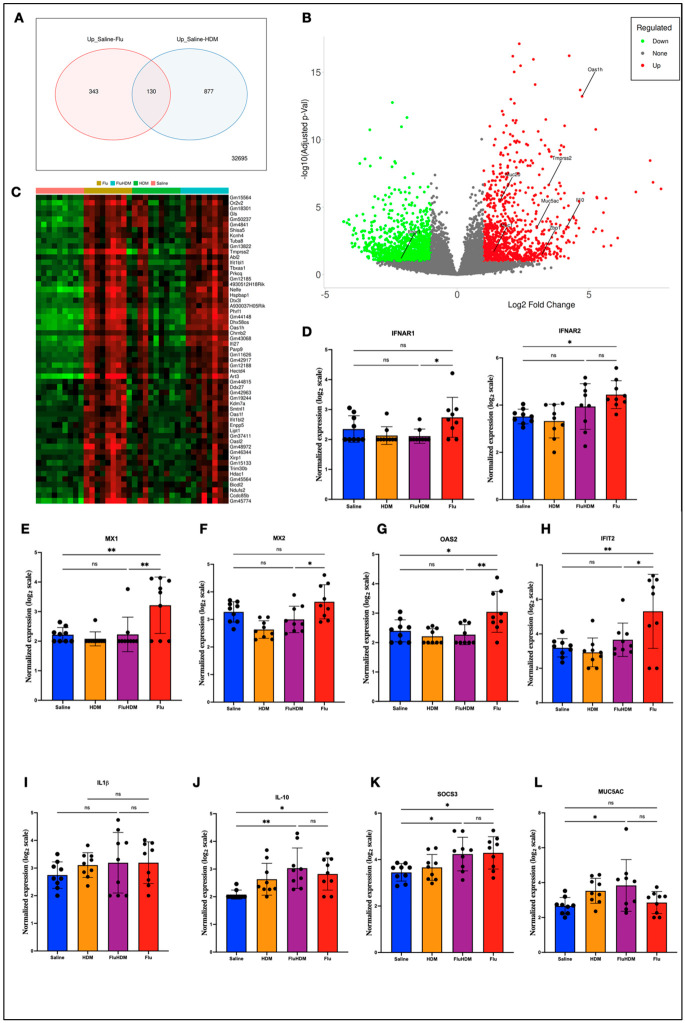
Immune responses mediated by influenza infection in mice with pre-existing HDM-induced allergic inflammation. (**A**) Venn diagram displaying overlap of significantly upregulated genes between Flu and HDM groups. (**B**) Volcano plot of differential upregulated genes (Flu+HDM vs. control); red = upregulated; green = downregulated; grey = not significant. Certain genes are annotated. (**C**) Heatmap of differentially expressed genes in the four experimental groups (Saline, Flu, HDM, and Flu+HDM). The heatmap provides a global overview of interferon-related and pro-inflammatory gene expression patterns and facilitates comparison of antiviral responses between groups, including antiviral genes (*Ifit1bl1*, *Ifi27*, *Oas1h*, *Oasl2*). Red and green indicate relatively higher and lower gene expression levels, respectively. (**D**) Bar graphs showing the normalized expression (log_2_ scale) of *IFNAR1* and *IFNAR2* genes across the groups. (**E**–**H**) Bar graphs showing the normalized gene expression (log_2_ scale) of ISGs *MX1* (**E**), *MX2* (**F**), *OAS2* (**G**), and *IFIT2* (**H**) across groups. (**I**–**L**) Bar graphs showing the normalized expression (log_2_ scale) of pro-inflammatory *IL1β* (**I**), *MUC5AC* (**L**) and regulatory genes *IL-10* (**J**), *SOCS3* (**K**) across groups. Data are represented as mean ± SD; ns, not significant; * *p* ≤ 0.05; ** *p* ≤ 0.01.

Six datasets were selected to investigate the effects of viral infections on asthma. A brief summary of each dataset is presented in Table 1. The flowchart in Figure 1 shows the dataset selection, preparation, and analysis. Viruses such as influenza and rhinovirus exacerbate asthma. In this study, house dust mites (HDM) were used as allergens to trigger airway inflammation in mice. Mice and rats are commonly used animal models to study asthma and chronic airway inflammation. To investigate how pre-existing allergic inflammation alters gene expression induced by influenza, we compared genes upregulated by influenza with those upregulated by HDM (Figure 2A). The Venn diagram shows an overlap of only 130 genes between the two groups. Influenza upregulated 343 genes in the lungs, whereas HDM exposure induced upregulation of 877 genes. This suggests that viruses and allergens induce distinct transcriptional profiles in the lungs. The volcano plot shows the upregulation (red) and downregulation (green) of genes in HDM-treated mice after influenza infection (Figure 2B). Several genes were upregulated, including antiviral genes, ISGs, and regulatory and functional epithelial-related genes, including *OAS1h*, *ZBP1*, *IL-10*, *MUC5AC*, and *TMPRSS2*. Certain genes, such as *IFNGR1*, were downregulated. A heatmap of the genes upregulated or downregulated in the experimental groups (Saline, Flu, HDM and Flu+HDM) is shown (Figure 2C). The heatmap shows the upregulation of specific genes, including interferon- and antiviral-related genes such as *IFI27*, *OAS1h*, and *OASL2* in Flu and Flu+HDM groups. To evaluate the role of interferon in mice with pre-allergic airway inflammation and viral infection, the expression of interferon receptor genes (*IFNAR1* and *IFNAR2*) in all groups was measured (Figure 2D). The expression of interferon receptors increased in the Flu-infected mice compared to that in the other groups (Saline, HDM and Flu+HDM). Notably, mice exposed to Flu+HDM had significantly lower interferon receptors than mice infected with Flu alone. This suggests impaired interferon transcriptional responses in the lungs of mice with allergic inflammation caused by HDM. Consistently, the expression of ISGs, including *MX1*, *MX2*, *OAS2*, *IFIT2*, *IFIT3*, *RSAD2*, and *ZBP1*, was statistically higher in the Flu-infected group compared to other groups (Figure 2E–H, Supplementary Figure S1).

*IL-1β* expression showed a slight, non-significant increase in all groups compared to that in the control group, suggesting the presence of modest inflammation (Figure 2I). Similarly, *IL-10* expression significantly increased in other groups compared to the control group, with the highest increase in the Flu+HDM group, suggesting regulatory responses (Figure 2J). In addition, *SOCS3* expression was significantly increased in the Flu+HDM and Flu groups compared to the saline group, suggesting activation of negative feedback mechanism that regulates cytokine signaling through JAK/STAT pathway (Figure 2K). Finally, the expression of *MUC5AC* significantly increased in the Flu+HDM group, causing an elevation of mucins, which is usually associated with an increased inflammatory airway (Figure 2L). Furthermore, Flu+HDM showed broad enrichment of immune-related pathways, including immune response, defense response, cytokine-cytokine receptor interaction, and antigen presentation pathways (Supplementary Figure S2). However, this activation of immune pathways was accompanied by comparatively weak expression of several interferon-related antiviral genes relative to Flu only group. Collectively, these results suggest that pre-allergic inflammation in mouse models of HDM-induced asthma reduces interferon and antiviral transcriptional immune responses in the presence of viral infection.

In another dataset, GSE157441, the effects of viral infection on asthma exacerbation were measured in asthma-susceptible Brown Norway (BN) rats. This model is typically used to study virus-induced asthma exacerbations in children characterized by the IRF7lo (low) phenotype. BN rats were divided into four experimental groups, as described in Table 1. The heatmap shows a distinctive pattern in the third group (virus+OVA2), with upregulation of a number of genes, including antiviral, pro-inflammatory, and regulatory genes (Supplementary Figure S3A). The ISGs (*MX1*, *ISG20*, *ZBP1*, and *IFIT3*) were significantly higher in the virus+OVA2 group than in the other groups (Supplementary Figure S3B–E). The expression of the regulatory and anti-inflammatory gene *TGF-β1* was also significantly increased in the virus+OVA2 group (Supplementary Figure S3F). Similarly, *IL-10* also increased (although not significantly) in the virus+OVA2 group compared to the other groups (Figure S3G). In addition, genes associated with airway inflammation, including *IL-6* (pro-inflammatory cytokine) and *SOCS3* (negative feedback regulator), were significantly upregulated in the virus+OVA2 group (Supplementary Figure S3H,I). Moreover, *TNFSF9*, which plays a role in airway inflammation and immune cell activation, was highly expressed in the virus+OVA2 group (Supplementary Figure S3J). MUC5B, which encodes one of the mucus components, was modestly increased in the virus+OVA2 group (Supplementary Figure S3K). Collectively, these results showed that antiviral, interferon, and immunoregulatory gene expression peaked at day 2 of virus/allergen exposure; however, this expression returned to baseline over time.

**Figure 3:**
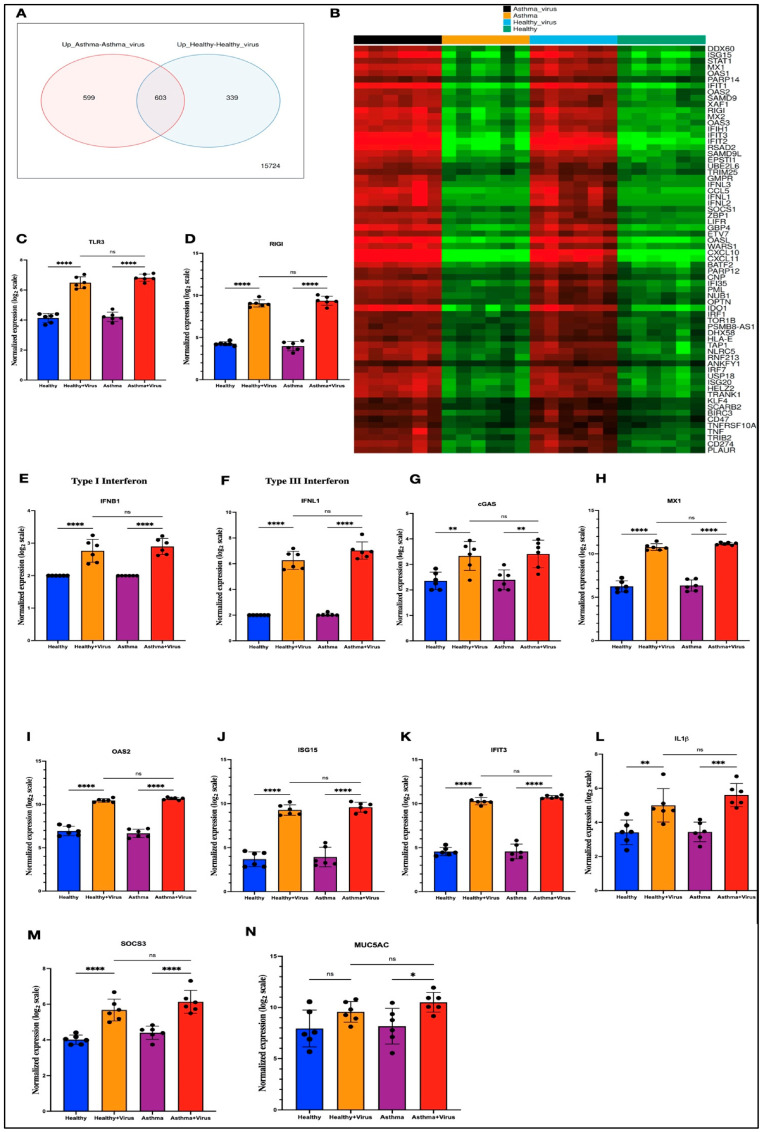
Immune responses mediated by healthy and asthmatic airway epithelial cells following rhinovirus infection. (**A**) Venn diagram showing the overlap of significantly upregulated genes between asthmatic and healthy airway epithelial cells after viral infection. (**B**) Heatmap of differentially expressed genes in airway epithelial cells across experimental groups (Asthma+virus, Asthma, Healthy+virus, and Healthy). The heatmap provides a global visualization of antiviral and inflammatory gene expression patterns and facilitates comparison of interferon transcriptional responses between healthy and asthmatic airway epithelial cells after viral infection, including interferon-related genes (*ISG15*, *MX1-2*, *IFIT1-3*, *OAS1-3*, *IFIH1*, *RIGI* and *CXCL10*). Red and green indicate relatively higher and lower gene expression levels, respectively. (**C**,**D**) Bar graphs showing the normalized expression (log_2_ scale) of *TLR3* (**C**) and *RIGI* (**D**) genes across the groups. (**E**,**F**) Bar graphs showing the normalized expression (log_2_ scale) of *IFNLB1* (**E**) and *IFNL1* (**F**) genes across the groups. (**G**–**K**) Bar graphs showing the normalized expression (log_2_ scale) of ISGs *cGAS* (**G**), *MX1* (**H**), *OAS2* (**I**), *ISG15* (**J**) and *IFIT3* (**K**) across groups. (**L**–**N**) Bar graphs showing the normalized expression (log_2_ scale) of pro-inflammatory *IL1β* (**L**), *MUC5AC* (**N**) and regulatory gene *SOCS3* (**M**) across groups. Data are represented as mean ± SD; ns, not significant; * *p* ≤ 0.05; ** *p* ≤ 0.01; *** *p* ≤ 0.001; **** *p* ≤ 0.0001.

To test whether human epithelial cells induce immune responses similar to those in lung tissues from animal models with pre-existing airway inflammation during viral infection, we investigated the effects of rhinovirus infection on human airway epithelial cells derived from asthmatics. The Venn diagram shows the overlap of a large set of genes—603 upregulated in airway epithelial cells from asthmatic and healthy subjects after viral infection (Figure 3A). A set of 599 genes was upregulated in epithelial cells from asthmatic patients after viral infection, and only 339 genes were upregulated in cells from healthy controls following viral infection. This indicates that rhinovirus infection induces a specific transcriptional profile in airway epithelial cells from asthmatics and healthy individuals, and numerous genes are shared between the two groups following infection. Heatmaps of differentially expressed genes in airway epithelial cells following rhinovirus infection showed similar patterns in both the asthma and healthy groups (Figure 3B). Many genes, including interferon-related genes (*DDX60*, *ISG15*, *ISG20*, *STAT1*, *MX1*, *OAS1-3*, *IFIT1-3*, *RSAD2*, *CXCL10*, *CCL5*, *OASL*, *IFNL1-3*, *ZBP1*, and *IFIH1*), were upregulated in both groups after viral infection. These results indicate that airway epithelial cells from healthy and asthmatic individuals induce similar immune responses to rhinovirus infection. Given that the rhinovirus is a positive-strand RNA virus that generates double-stranded RNA (dsRNA) intermediates during replication, we investigated the expression of dsRNA sensors. TLR3 and RIG-I are critical dsRNA sensors that are activated during rhinovirus infection. The expression of these sensors significantly increased in both asthmatic and healthy epithelial cells after rhinovirus infection (Figure 3C,D). The baseline expression for both sensors did not differ between the groups, and no significant difference was observed in the asthmatic and healthy epithelial cells after viral infection. Rhinovirus infection induced a significant increase in the expression of Type I (*IFNB1*) and Type III (*IFNL1*) in airway epithelial cells from both asthmatic and healthy individuals compared to the baseline levels (Figure 3E,F). ISGs, including (*cGAS*, *MX1*, *OAS2*, *ISG15*, *IFIT3*, *OAS1*, *OAS3*, *RSAD2*, and others) were significantly increased in both groups after viral infection (Figure 3G–K, Supplementary Figure S4). Both groups had comparable baseline levels before rhinovirus infection. Similarly, after infection, the level of increase in ISGs induction was comparable in both the groups. Additionally, genes involved in antiviral interferon response included *IFNGR2*, *CXCL10*, *STAT1*, *IRF7*, *IRF9*, and *CCL5*, followed a similar expression pattern (Supplementary Figure S5).

To determine the level of inflammation in the groups after viral infection, we measured pro-inflammatory cytokine *IL-1β* expression. Rhinovirus infection increased *IL-1β* expression in both groups, although the asthma group had slightly higher *IL-1β* induction compared to the virus-infected healthy cells (Figure 3L). Similarly, the induction of *SOCS3* increased in both groups after viral infection (Figure 3M). Lastly, *MUC5AC* expression increased in both asthmatic and healthy epithelial cells compared with baseline; however, statistical significance was only observed in the asthmatic group (Figure 3N). Similarly, *MUC5AC* expression also increased in healthy epithelial cells; however, the difference was not significant. Together, these results suggest that airway epithelial cells from asthmatic and healthy individuals induce similar immune responses related to viral sensing, interferon preservation, inflammatory responses, and, to some extent, mucus production.

**Figure 4:**
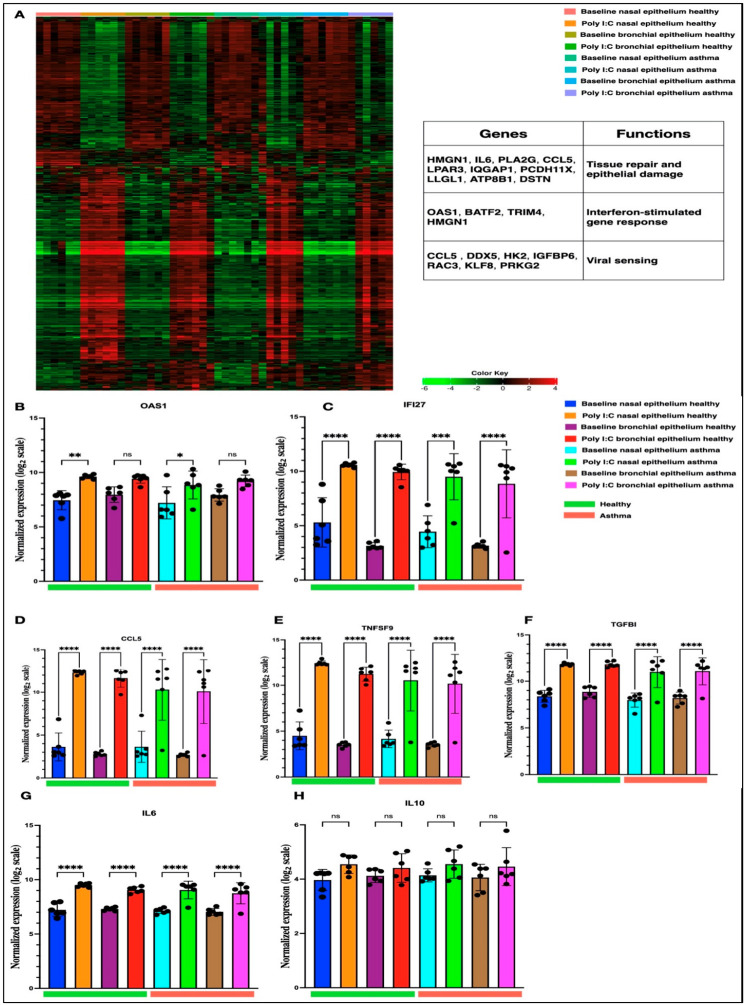
Transcriptional immune responses of nasal and bronchial epithelial cells after Poly I:C stimulation. (**A**) Heatmap of differentially expressed genes in nasal and bronchial epithelial cells before and after Poly I:C stimulation across experimental groups (Healthy nasal epithelial cells, Healthy bronchial epithelial cells, Asthmatic nasal epithelial cells, and Asthmatic bronchial epithelial cells). Gene expression is shown on a red-to-green scale, with red denoting higher expression and green denoting lower expression. The heatmap provides a visualization of gene expression related to interferon, tissue repair and epithelial damage across epithelial cell types. The table lists selected genes with their functions. (**B**,**C**) Bar graphs showing the normalized expression (log_2_ scale) of ISGs *OAS1* (**B**) and *IFI27* (**C**) across the groups. (**D**–**H**) Bar graphs showing the normalized expression (log_2_ scale) of pro-inflammatory genes *CCL5* (**D**), *TNFSF9* (**E**), and *IL6* (**G**), airway remodeling-associated gene *TGFBI* (**F**), and regulatory gene *IL10* (**H**) across experimental groups before and after Poly I:C stimulation. Data are represented as mean ± SD; ns, not significant; * *p* ≤ 0.05; ** *p* ≤ 0.01; *** *p* ≤ 0.001; **** *p* ≤ 0.0001.

Next, we investigated the effects of viral infections on nasal and bronchial epithelial cells from asthmatic and healthy donors. Poly I:C is a synthetic double-stranded RNA (dsRNA) that mimics viral infection. According to data from the dataset GSE51392, nasal and bronchial epithelial cells were stimulated with Poly I:C. A heatmap of the differentially expressed genes in both nasal and bronchial epithelial cells before and after Poly I:C stimulation is shown (Figure 4A). After Poly I:C stimulation, a number of genes were expressed, including genes related to tissue repair, epithelial damage, interferon response, and viral sensing. Poly I:C stimulation induced gene expression of *OAS1* in the nasal epithelium and *IFI27* in the nasal and bronchial epithelium of both healthy and asthmatic subjects (Figure 4B,C). *CCL5* was significantly increased after Poly I:C stimulation in nasal and bronchial epithelial cells from healthy and asthmatic subjects, suggesting increased immune cell recruitment (Figure 4D). Similarly, *TNFSF9* levels also increased after Poly I:C stimulation, suggesting elevated immune costimulatory signaling in nasal and bronchial cells from both groups (Figure 4E). In addition, *TGFBI* significantly increased after stimulation with Poly I:C, indicating increased epithelial tissue repair and remodeling (Figure 4F). The pro-inflammatory cytokine *IL-6* also increased following Poly I:C treatment in both groups, suggesting an active state of inflammation and immune signaling (Figure 4G). Moreover, *IL-10* levels also increased after Poly I:C stimulation; however, the difference was not statistically significant (Figure 4H). Finally, *SERPING1* (encoding a C1 esterase inhibitor), which plays a role in complement regulation and inflammation inhibition, was significantly upregulated in nasal and bronchial epithelial cells from healthy and asthmatic donors after Poly I:C stimulation (Supplementary Figure S6). Collectively, these results suggest that nasal and bronchial epithelial cells from asthmatics behave similarly to cells from healthy donors after viral-like stimulation, suggesting that antiviral and interferon-related transcriptional immune responses are preserved.

**Figure 5:**
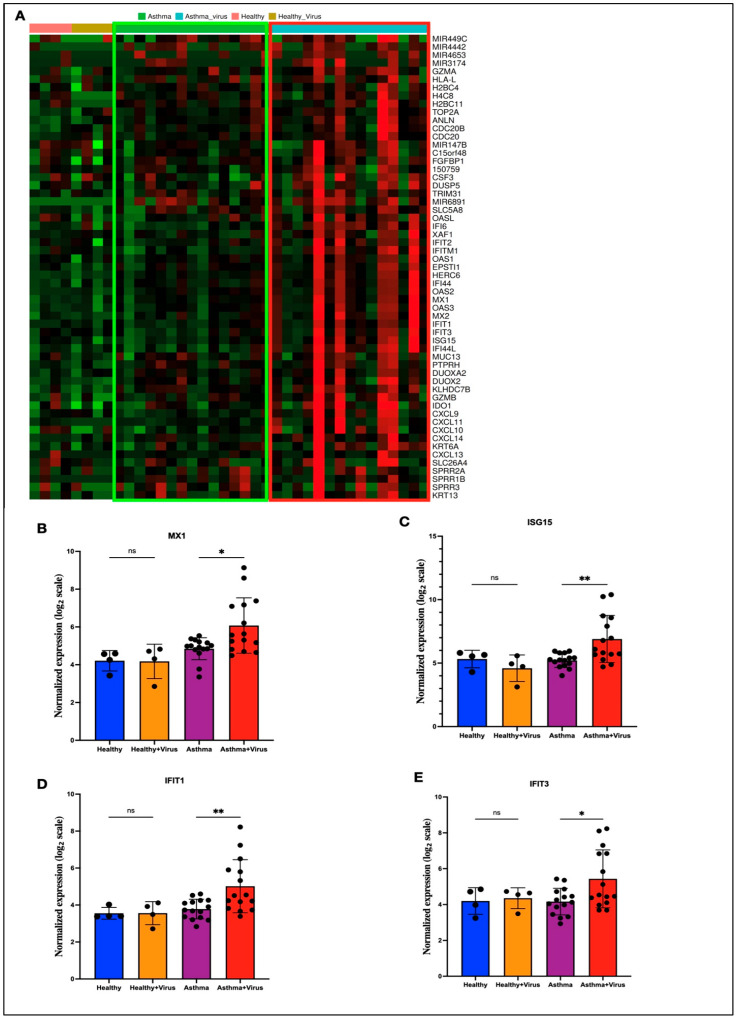
Expression of ISGs following RV16 infection in healthy and asthmatic PBECs. (**A**) Heatmap of differentially expressed genes in PBECs obtained from healthy and asthma donors before and after RV16 challenge. The heatmap provides an overview of antiviral and inflammatory transcriptional responses following RV16 infection, including (*MX1-2*, *IFIT1-2*, *OAS1-3*, *ISG15* and *CXCL10*). Red and green indicate relatively higher and lower gene expression levels, respectively. (**B**–**E**) Bar graphs showing normalized expression (log_2_ scale) of the ISGs *MX1* (**B**), *ISG15* (**C**), *IFIT1* (**D**), and *IFIT3* (**E**) in PBECs from the following groups (Healthy, Healthy+Virus, Asthma, and Asthma+Virus). Data are represented as mean ± SD; ns, not significant; * *p* ≤ 0.05; ** *p* ≤ 0.01.

To further confirm the preservation of interferon immune responses in asthmatics, we examined the expression of interferon-related genes, including ISGs. Here, PBECs were isolated from healthy individuals (n = 4) and patients with mild asthma (n = 15) before and after 6 days of *in vivo* RV16 challenge. The heatmap shows differential gene expression in both healthy and asmatic individuals before and after the RV16 challenge (Figure 5A). The heatmap shows high upregulation of ISGs, including *OAS1–3*, *MX1*, *MX2*, *IFIT1–3*, *ISG15*, *IFI6*, *IFI44*, *IFI44L*, and *OASL*, in the asthmatic group; however, this trend was not observed in the healthy group, most likely due to the limited sample size (n = 4). *MX1*, *ISG15*, *IFIT1*, and *IFIT3* were significantly increased in PBECs of asthma patients after RV16 viral infection (Figure 5B–E). In addition, the expression of many ISGs, including *RSAD2*, *ISG20*, and others, was higher in the asthma group than in the healthy group after the viral challenge, although the differences did not reach statistical significance (Supplementary Figure S7). Some genes related to viral sensing (*RIG-I* and *TLR3*), interferon signaling (*STAT1* and *IRF7*), and complement regulation (*SERPING1*) slightly increased in the asthma group after viral infection; however, this increase was not statistically significant (Supplementary Figure S8). Together, these results confirm the preservation of virus-induced antiviral immune responses in the bronchial epithelial cells of asthmatic individuals.

**Figure 6:**
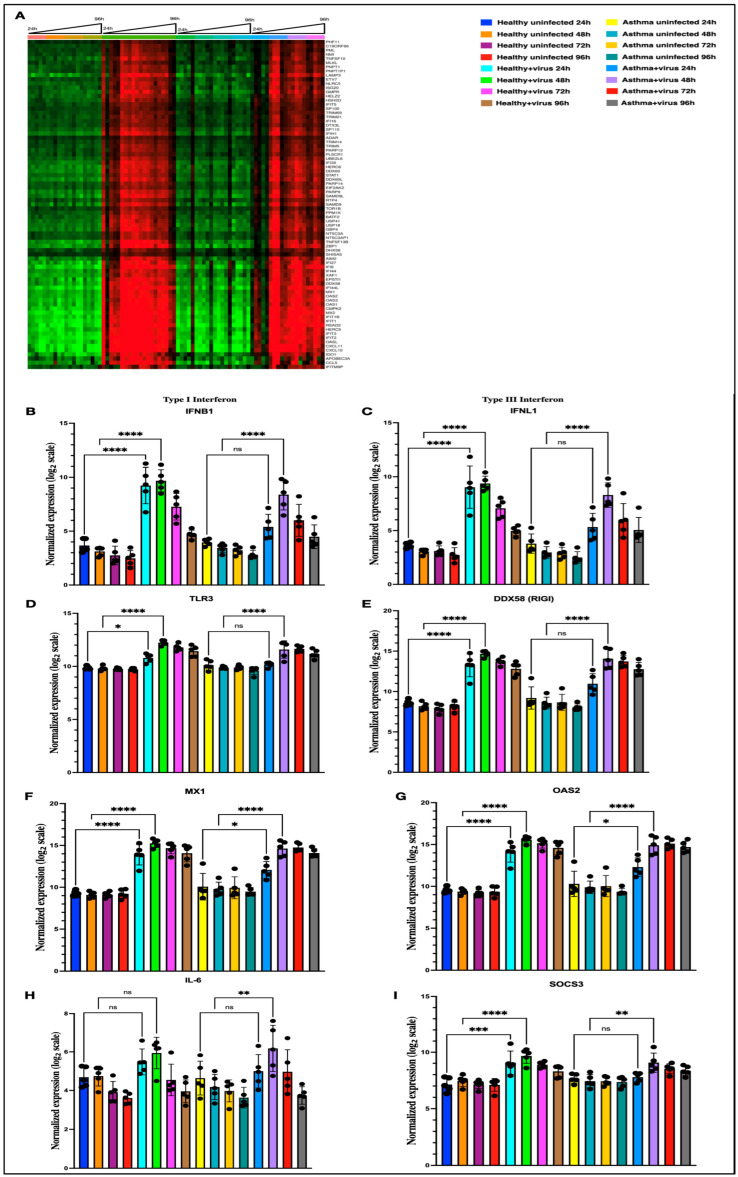
Time-dependent antiviral and interferon-related gene expression (24–96 h) following rhinovirus 1A infection in primary epithelial cells from healthy and asthmatic subjects. (**A**) Heatmap of differentially expressed genes in primary epithelial cells obtained from healthy and asthma donors before and after rhinovirus 1A challenge. The heatmap provides a global overview of antiviral gene expression patterns and facilitates evaluation of interferon responses in healthy and asthmatic epithelial cells after viral infection, including (*MX1-2*, *IFIT1-3*, *OAS1-3*, *CXCL10-11*). Red and green indicate relatively higher and lower gene expression levels, respectively. (**B**,**C**) Bar graphs showing the normalized expression (log_2_ scale) of *IFNB1* (**B**) and *IFNL1* (**C**) genes across the groups (Healthy uninfected 24–96 h, Healthy+virus 24–96 h, Asthma uninfected 24–96 h, and Asthma+virus 24–96 h). (**D**,**E**) Bar graphs showing the normalized expression (log_2_ scale) of the dsRNA sensors *TLR3* (**D**) and *RIGI* (**E**) genes across the groups before and after rhinovirus 1A challenge. (**F**,**G**) Bar graphs showing normalized expression (log_2_ scale) of the ISGs *MX1* (**F**), and *OAS2* (**G**) across groups before and after rhinovirus 1A challenge. (**H**,**I**) Bar graphs showing the normalized expression (log_2_ scale) of pro-inflammatory *IL6* (**H**), and regulatory gene *SOCS3* (**I**) across groups before and after rhinovirus 1A challenge. Data are represented as mean ± SD; ns, not significant; * *p* ≤ 0.05; ** *p* ≤ 0.01; *** *p* ≤ 0.001; **** *p* ≤ 0.0001.

Moreover, the preservation of the interferon-related immune response and innate sensor signaling in primary epithelial cells obtained from asthma patients was tested at different time points (24, 48, 72, and 96 h). Cells were challenged with rhinovirus 1A at the indicated time points and transcriptional gene expression was measured. After viral infection of primary epithelial cells with rhinovirus 1A, the transcriptional pattern was similar between cells from healthy and asthmatic donors (Figure 6A). Virus-induced gene expression in the asthma group was observed only after 48 h of infection when compared to healthy controls, which were active 24 h post-infection. Type I interferon (*IFNB1*) and Type III interferon (*IFNL1*) genes were highly upregulated (*p* < 0.05,) after 24 h and 48 h of rhinovirus 1A infection in cells from healthy donors (Figure 6B,C). However, there was no significant change in the expression of *IFNB1* and *IFNL1* genes after 24 h of Rhinovirus 1A infection in the asthma group (Figure 6B,C). After 48 h, the expression of *IFNB1* and *IFNL1* in both (asthma and healthy) groups was similar. This suggests that the interferon transcriptional responses are present in both healthy and asthmatic primary epithelial cells; however, there was a 24 h delay in the asthma group. *TLR3* expression significantly increased after 24 h but reached maximal expression after 48 h of infection in the healthy group (Figure 6D). However, in asthmatic cells, viral infection induced *TLR3* expression only after 48 h with no significance observed after 24 h (Figure 6D). Similarly, the expression of *RIG-I* was observed after 24 h and 48 h of infection only in the healthy group, whereas, in the asthma group, the expression of *RIG-I* was significantly increased after 48 h of viral infection (Figure 6E). This suggests that the viral dsRNA sensing response is delayed in the asthma group compared to that in the control group. ISGs, including *MX1* and *OAS2*, were highly upregulated by rhinovirus 1A infection in the healthy group after 24 h and 48 h (Figure 6F,G). In the asthma group, *MX1* and *OAS2* upregulation after viral infection was significantly but modestly increased after 24 h (*p* < 0.05); however, after 48h, the expression of ISGs was similar to that in the healthy group (*p* < 0.05) (Figure 6F,G). The same pattern was also observed with other ISGs, including *IFIT1*, *IFIT3*, *MX2*, and others (Supplementary Figures S9 and S10). The gene encoding for the pro-inflammatory cytokine *IL-6* increased in both groups after 24 h and 48 h of viral stimulation; however, it only reached statistical significance after 48 h of stimulation in the asthma group, suggesting increased airway inflammation compared to the healthy group (Figure 6H). In addition, *SOCS3* expression significantly increased after 24 h and 48 h of infection in healthy cells, whereas in asthmatic cells, significant increase was only observed after 48 h of viral infection (Figure 6I). This suggests induction of negative feedback regulator SOCS3, which inhibits JAK/STAT-mediated interferon signaling and limits excessive inflammatory response in both the healthy and asthma groups, although the induction appears to be delayed in the asthmatic group.

To further validate our findings, we analyzed an independent RNA-sequencing dataset (GSE149273) comprising primary airway epithelial cells from asthmatics before and after viral infection with rhinovirus. Viral infection resulted in significant upregulation of type I and type III interferons and several ISGs including *IFNB1*, *IFNL1*, *IFIT1-3*, *OAS1-3*, *ISG15*, *MX1*, *MX2*, *CCL5*, *CXCL10*, *TLR3* and *MDA5*. These results provide additional confirmation for the preservation of antiviral interferon transcriptional responses in asthmatic airway epithelial cells.

## 3. Discussion

Asthma is a complex disorder affected by various environmental and genetic factors. The airway epithelium is the first line of defense against microbes, including viruses, bacteria, and inhaled allergens. The airway epithelium plays an important role in allergen-induced type 2 lung inflammation. Allergens trigger airway epithelial cells to release important cytokines (IL-25, IL-33, and TSLP). These cytokines trigger ILC2s to produce IL-4, IL-5, and IL-13, which play significant roles in orchestrating the symptoms observed in asthmatic individuals. Such symptoms include eosinophilia, increased mucus production by goblet cells, lung tissue remodeling, and overall airway hyper-responsiveness. Viral-induced exacerbations in asthma have been linked to epithelial layer impairment and leakage. Reduced tight junction proteins (e.g., claudins and occludin) allow allergens to penetrate the epithelium layer and directly contact immune cells, such as ILC2s and dendritic cells, which enhance asthma exacerbation [8,9,32,33]. Studies have reported conflicting results regarding the functionality of the epithelium in asthma. Some studies report that airway epithelial cells induce low type I/III interferons in response to viruses, whereas others have demonstrated that cells induce an interferon response, but this induction is delayed [18,23,34]. In this study, we aimed to determine whether the antiviral interferon response is impaired in the asthmatic airway epithelium. Several studies investigating asthma exacerbations have relied mostly on findings from murine animal models. Here, we explored the effects of viral infections in different asthma models (mice and rats), as well as in humans, to assess the impact of viruses on airway inflammation. Our results demonstrate that, in animal models of asthma (mice and rats), the interferon response was significantly reduced after viral infection. However, in humans, no statistical difference was observed in the interferon-related responses between asthmatics and healthy controls following viral infection. One reason for this inconsistency between species could be the type of tissue being analyzed for transcriptional profiling, specifically the lung tissue in animal models versus airway epithelial cells in humans. Additionally, the induction of interferon-related responses in nasal and bronchial cells was similar in both asthmatic and healthy subjects following Poly I:C stimulation.

Although several studies have reported that patients with asthma exhibit deficient or impaired interferon response to viral infection, our findings suggest that the interferon transcriptional responses are preserved in the airway epithelium of asthmatics when compared to healthy controls during viral infections. These observations align with recent findings reported by Veerati et al. (2020), where the interferon response triggered by viral infection was delayed rather than deficient [23]. In addition to the interferon transcriptional response, the expression of several genes associated with asthma (*MUC5AC*, *MUC5B*), viral sensing (*TLR3*, *RIGI*), and inflammation (*CCL5*, *TNFSF9*, *IL-6*, *IL1β*) did not significantly differ between asthmatic and healthy subjects following viral infection. Moreover, the heatmaps showed similar expression patterns between healthy and asthmatic epithelial cells following viral infection, with no evidence of impaired interferon response in the asthma group. These findings suggest that antiviral transcriptional responses are preserved in human asthmatic airway epithelial cells.

While several studies have suggested that the interferon response is impaired in asthma, our results support the notion that epithelial cells are able to mount robust antiviral interferon responses. Strong evidence suggests that a dysfunctional epithelium barrier and weak tight junctions, rather than defects in interferon production, may increase the susceptibility to viral infection, asthma exacerbations and allergen exposure [35,36]. Studies have showed that Claudin-18, one of the major lung specific tight junction proteins is deficient in asthmatics [24,37,38,39]. Epithelial brushings from asthmatics have significantly lower *CLDN18* mRNA levels compared to healthy controls [37]. This reduction in tight junction and adherens junction proteins, including (Claudin-18, occluding and ZO1) may facilitate virus and allergen entry through the epithelium, thus, contributing to asthma exacerbations [38]. In addition, the delayed interferon response in asthmatic airway epithelium facilitates viral replication and dissemination, thereby contributing to asthma exacerbations.

A key limitation is that our findings rely only on publicly available microarray/RNA-seq datasets without experimental validation. In addition, interferon induction through mRNA levels without protein quantification is considered a limitation of this study. Future studies should include the assessment of protein in addition to mRNA level measurements to validate the observed transcriptional patterns observed in asthmatics after respiratory viral infections. In addition, one of the limitations of this study is the small sample size of the healthy groups in Figure 5 (n = 4 per group), which may have reduced statistical power and limited the detection of statistical differences in the healthy group. Also, most of the datasets included in this study lacked sufficient clinical information to allow stratification by asthma phenotype or endotype and disease severity. Future studies should include characterized asthma cohorts with known phenotypes to determine whether interferon responses to viral infections differ among specific asthma subgroups. Of note, although standardized preprocessing and analyses were performed for each dataset independently, GEO datasets are heterogeneous in regard to experimental protocols, platforms, and availability of metadata. Nonetheless, our conclusions were based on reproducible findings observed across several independent datasets.

## 4. Materials and Methods

### 4.1. Gene Expression Data Acquisition

Expression datasets were downloaded from the Gene Expression Omnibus (GEO) database (https://www.ncbi.nlm.nih.gov/geo/ (accessed on 3 November 2025)). Six independent datasets were used in this study, consisting of microarray and RNA sequencing data from mice, rats, and human models. Gene expression was investigated in the lung tissues of animal models and in airway nasal and bronchial epithelial cells in humans.

The GSE293097 dataset contains RNA sequencing data derived from the lung tissue of mice with pre-existing allergic inflammation following viral infection. Mice were intranasally administered saline or house dust mite (HDM) allergen extract three times per week for 24 days, followed by influenza A virus (H3N2 X31) infection. The groups were divided as follows: 1—saline/saline, 2—HDM/saline, 3—saline/influenza, 4—HDM/influenza groups. Lung tissues were collected three days post-infection for RNA-sequencing analysis.

GSE157441 consists of RNA-sequencing data from the lung tissues of Rattus norvegicus (asthma-susceptible Brown Norway (BN) rats). Rats were sensitized to ovalbumin (OVA)/alum, infected with a murine-adapted rhinovirus model (vMC0), and challenged with OVA. Samples were collected at different time points post-infection (1, 2, and 9 d post-infection). The experimental groups (n = 5–7) were divided as follows: 1, no OVA or virus (control); 2, no OVA with virus 1 day post-infection; 3, OVA and virus 2 days post-infection; and 4, OVA and virus 9 days post-infection. RNA-seq was performed on the lung tissues at the indicated time points.

GSE61141 includes RNA-seq data from differentiated air–liquid interface (ALI) cultured human airway epithelial cells obtained from six asthmatic and six non-asthmatic subjects. Cells were infected with human rhinovirus (HRV) or treated with vehicle control for 24 h, and RNA-seq was performed.

GSE51392 contains microarray data from primary nasal and bronchial epithelial cells isolated from 12 human subjects (6 allergic asthma subjects and 6 healthy controls). Epithelial cells were stimulated for 24 h with Poly I:C, which is a synthetic double-stranded RNA used as a viral mimic. Gene expression analysis was performed using a microarray (Affymetrix U133+ PM GeneChip Array).

The GSE106388 dataset included RNA-seq data from PBECs obtained from patients with mild asthma (n = 15) and healthy controls (n = 4). Cells were obtained by bronchial brushes before and after 6 days of *in vivo* RV16 challenge. Transcriptomic analysis was conducted to assess the interferon-related responses.

GSE146532 contains RNA-seq data of PBECs derived from healthy (n = 40) and asthmatic adults (n = 40) infected with rhinovirus 1A. Cells were infected at a low multiplicity of infection at four time points (24, 48, 72 and 96 h). Virus-induced interferon-related responses were assessed using RNA-seq.

### 4.2. Dataset Selection and Inclusion Criteria

Transcriptomic datasets were included only if they met these criteria: (1) contained clear experimental design, appropriate controls and experimental groups; (2) evaluated responses to viral infection in human airway epithelial cells or animal respiratory tissues; (3) included processed expression data or raw sequencing data suitable for downstream analysis. Based on these criteria, six datasets were selected, including five RNA-sequencing datasets (GSE293097, GSE157441, GSE61141, GSE106388, and GSE146532) and one microarray dataset (GSE51392).

### 4.3. Data Preprocessing and Normalization

Each dataset was analyzed independently without merging with other datasets. RNA-sequencing datasets were processed and analyzed using iDEP v2.4.4. For the microarray datasets, the normalized expression matrix provided by GEO was used and gene annotations were obtained using the corresponding platform annotation files. Differential expression and visualization analyses were subsequently performed separately for each dataset using standardized preprocessing procedures implemented within iDEP v2.4.4.

### 4.4. iDEP

Datasets containing processed and normalized gene expression data were obtained from the GEO database. Data were uploaded into iDEP v2.4.4 (South Dakota State University, Brookings, SD, USA) (https://bioinformatics.sdstate.edu/idep/ (accessed on 3 November 2025)) [40]. Gene expression analysis was performed using iDEP software v2.4.4. Genes were filtered according to the threshold levels implemented within iDEP v2.4.4. Gene expression data were exported from iDEP as CSV files, and statistical tests were performed using the GraphPad Prism Software 10.3.1 (GraphPad Software, Boston, MA, USA). Statistical differences among the experimental groups were determined using analysis of variance (ANOVA). Figures were generated using GraphPad Prism Software. Venn analysis, GO, and KEGG enrichment analyses of DEGs were performed using iDEP v2.4.4.

### 4.5. Statistical Analysis

Statistical analyses were performed using GraphPad Prism Software. Statistical differences between groups were analyzed using one-way or two-way ANOVA as appropriate. Statistical significance was set at *p* < 0.05. Data are expressed as mean ± SD.

A glossary of the major interferon-related, inflammatory, and epithelial function-associated genes discussed in this study are provided in (Supplementary Table S1). The table includes gene full names, categories, and biological functions relevant to antiviral immunity and asthma exacerbations.

## 5. Conclusions

In conclusion, our findings suggest that the interferon-associated transcriptional responses are preserved in asthmatic epithelial cells during viral infection. Nasal and bronchial epithelial cells from healthy and asthmatic subjects induced similar interferon-related responses following viral stimulation. Future studies should focus on the mechanisms associated with asthma exacerbation during viral infections.

## Figures and Tables

**Table 1:** Summary of the datasets used in the study.

Dataset	Brief Summary	Reference
GSE293097 RNA-seq	The study investigated how pre-existing allergic airway inflammation affects immune responses to viral infections. Mice were exposed to saline or HDM followed by nasal administration of influenza A or saline on day 21. Exposure to HDM resulted in high HDM-specific IgE, IgG1, IgG2, eosinophils, neutrophils, Th1, and Th17 cells compared to controls (saline). HDM and influenza co-infection showed reduced Th1 and regulatory T cells and increased Th2 cells. This may explain the increased susceptibility of asthma patients to viral infections.	[27]
GSE157441 RNA-seq	This study explores the immunological hallmarks and innate immune responses in children with virus-induced severe asthma. Virus-induced severe asthma exacerbation is characterized by IRF7hi and IRF7lo phenotypes. The animal models used in this study are asthma-resistant PVG, which represents IRF7hi, and asthma-susceptible BN rats, which represents IRF7lo phenotypes. RNA-seq was performed for lung tissues after viral and allergen exposure. Samples were divided into four groups (with n = 5–7); group 1 = no OVA or virus (saline), group 2 = no OVA with virus 1 day post-infection (DPI), group 3 = OVA and virus 2 DPI, group 4 = OVA and virus 9 DPI.	[28]
GSE61141 RNA-seq	The study investigates immune phenotypic responses of air–liquid interface (ALI) human airway epithelial cells to rhinovirus. In this study, airway epithelial cells were isolated from 6 asthmatic and 6 non-asthmatic donors then treated with either human rhinovirus (HRV) or vehicle control.	[29]
GSE51392 Microarray	In this study, upper (nasal) and lower (bronchial) airway epithelial cells were isolated from patients with allergic asthma and healthy controls. Cells were stimulated with Poly I:C, a synthetic analog of viral dsRNA, for 24 h, and gene expression was measured using microarray analysis.	[30]
GSE106388 RNA-seq	In this study, mild asthma patients (n = 15) and healthy controls (n = 4) were infected with RV16. Primary bronchial epithelial cells (PBECs) were obtained by bronchial brushes before and after 6 days of *in vivo* RV16 challenge.	[31]
GSE146532 RNA-seq	In this study, primary epithelial cells obtained from healthy controls and asthma patients were challenged with rhinovirus 1A at four different timepoints (24, 48, 72, and 96 h). Gene expression of interferons, interferon regulatory factors, TLR signaling, and transcription factors were measured at the indicated timepoints.	[23]

## Data Availability

All the datasets included in this study are publicly available in the GEO database (http://www.ncbi.nlm.nih.gov/geo (accessed on 3 November 2025)).

## Supplementary Materials

The following supporting information can be downloaded at: https://www.mdpi.com/article/10.3390/ijms27146113/s1.
