# Supplementary material for: Interferon-Associated Transcriptional Responses Are Preserved in Human Asthmatic Airway Epithelial Cells During Viral Infection
Source: Int J Mol Sci. 2026 Jul 8;27(14):6113. doi: 10.3390/ijms27146113 (PMC13410682; doi:10.3390/ijms27146113)
Supplement: Supplementary file 1 [file ijms-27-06113-s001.zip › ijms-4355916-supplementary.pdf]

Supplementary Materials

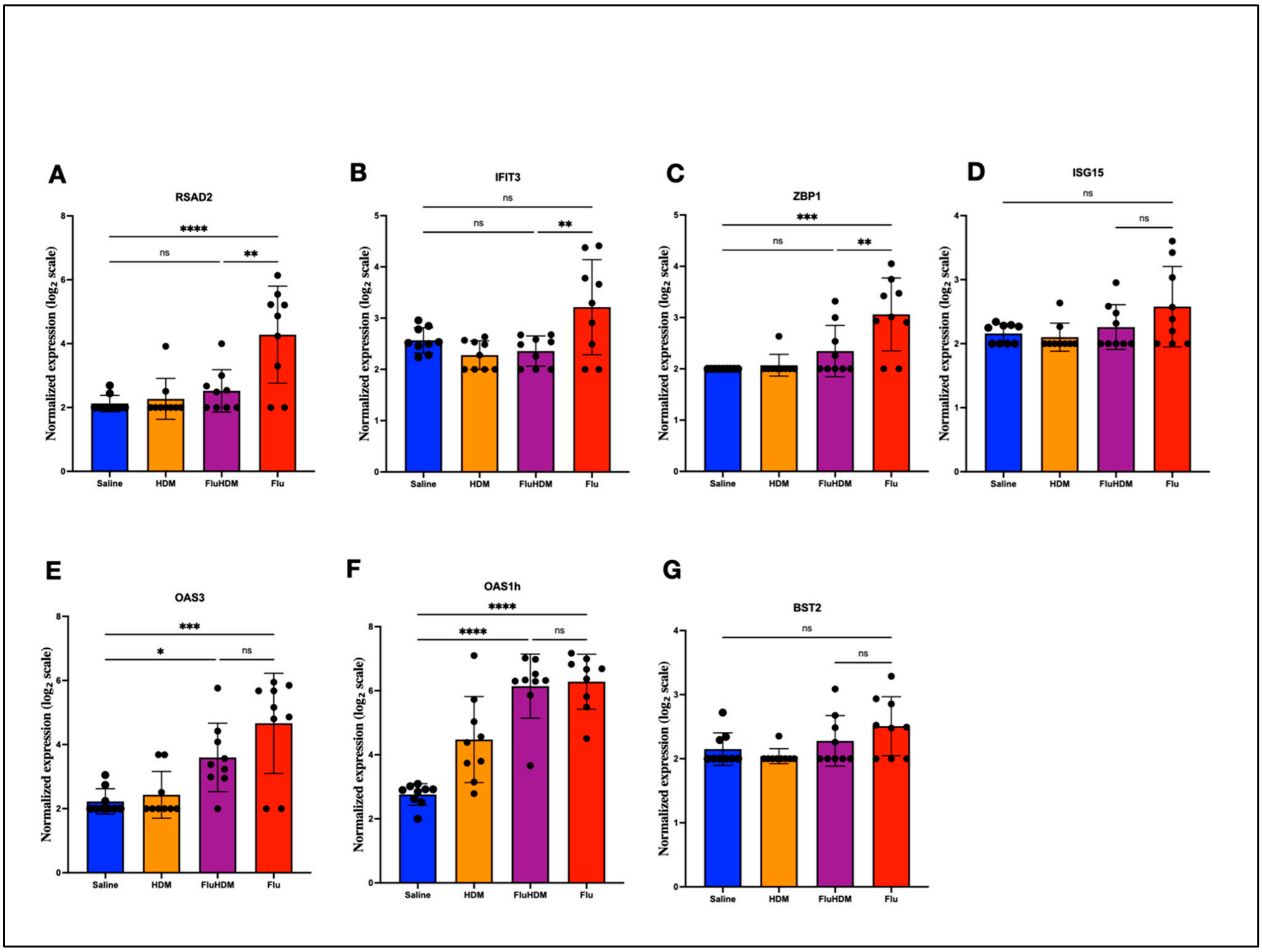

Figure S1. Immune responses mediated by influenza infection in mice with pre-existing HDM-induced allergic inflammation. (A–G) Bar graphs showing the normalized gene expression (log_2 scale) of interferon-stimulated genes RSAD2 (A), IFIT3 (B), ZBP1 (C), ISG15 (D), OAS3 (E), OAS1h (F), and BST2 (G) across the four experimental groups (Saline, Flu, HDM, and Flu+HDM). Data are represented as mean ± SD; ns, not significant; *P ≤ 0.05.

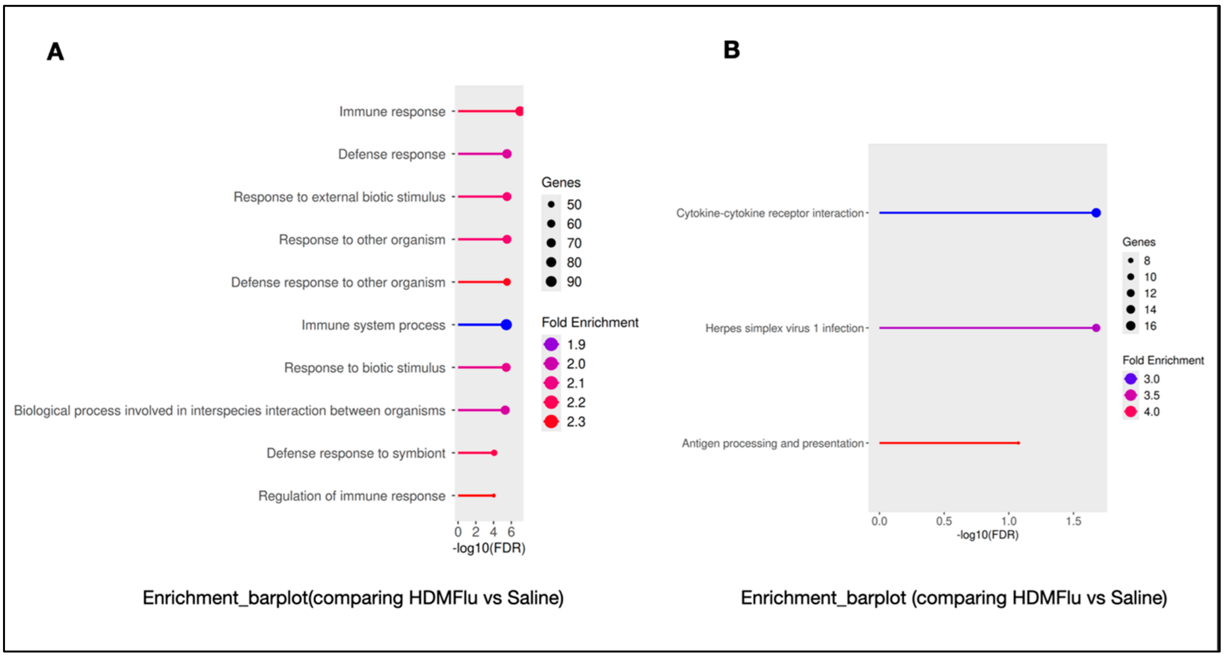

Figure S2. GO Enrichment analysis of expressed genes in HDM+Flu group compared to saline (control). (A) GO biological process enrichment analysis showing the top significantly enriched genes (−log10 FDR) in the HDM+Flu group compared to control. Dot size denotes the number of genes associated with each category; the color denotes the fold change. The analysis involved genes associated with a number of categories including immune responses, defense responses, and others. (B) KEGG pathway enrichment analysis of the same gene set using the same groups showing involvement of cytokine–cytokine receptor interaction, herpes simplex virus 1 infection, and antigen processing and presentation.

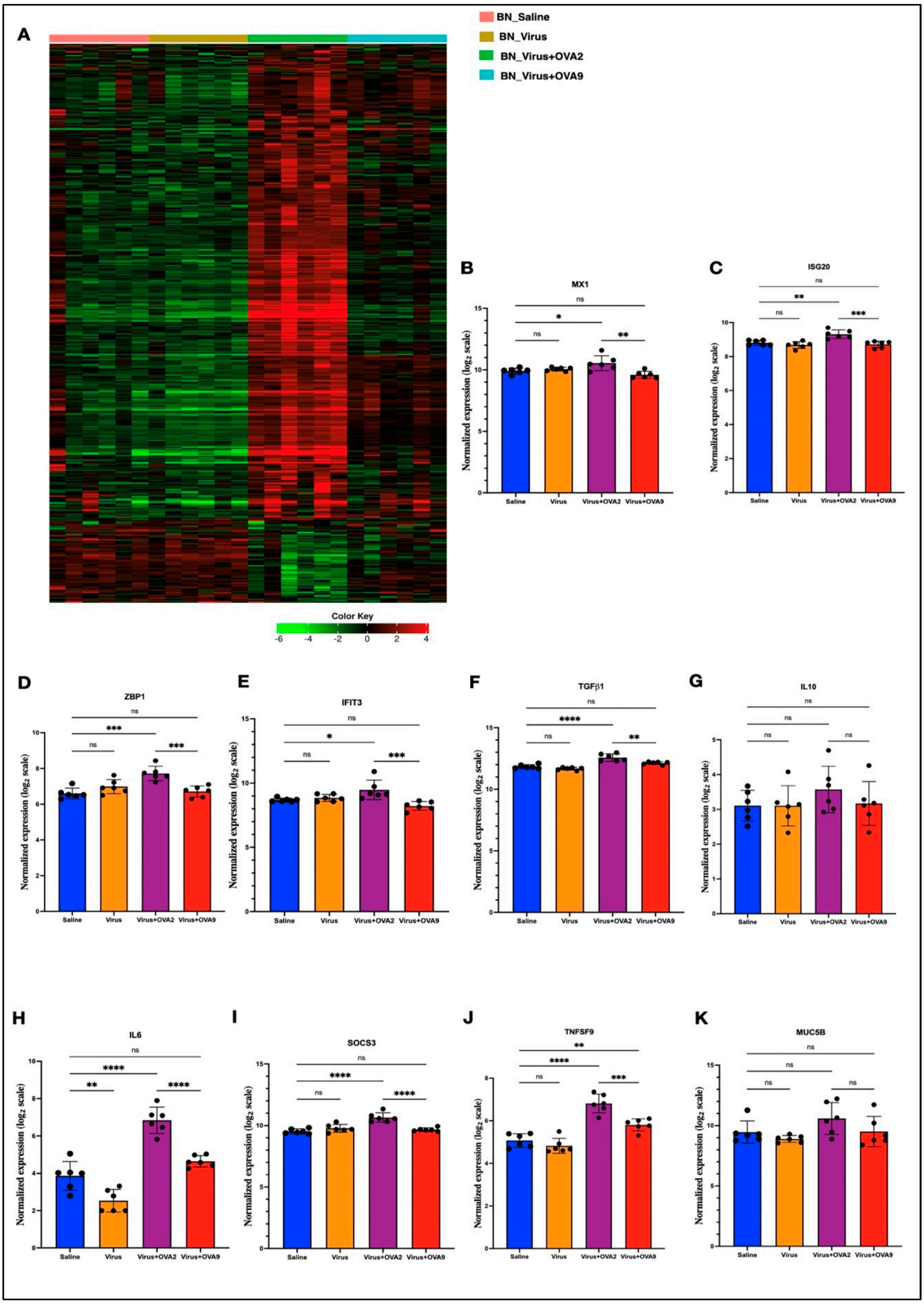
A
BN_Saline
BN_Virus
BN_Virus+OVA2
BN_Virus+OVA9
Color Key
-6 -4 -2 0 2 4
B
MX1
C
ISG20
D
ZBP1
E
IFIT3
F
TGFβ1
G
IL10
H
IL6
I
SOCS3
J
TNFSF9
K
MUC5B
Normalized expression (log2 scale)
Saline
Virus
Virus+OVA2
Virus+OVA9
ns
*
**
***
****

Figure S3. Immune responses mediated by virus/allergen exposure in asthma-susceptible BN rats. (A) Heatmap of differentially expressed genes in the lungs across experimental groups (group 1: saline, group 2: virus 1 day after infection, group 3: OVA and virus 2 days after infection, group 4: OVA and virus 9 days after infection). (B-E) Bar graphs showing the normalized expression (log_2 scale) of interferon-stimulated genes MX1 (B), ISG20 (C), ZBP1 (D), and IFIT3 (E) across groups. (F-G) Bar graphs showing the normalized expression (log_2 scale) of regulatory cytokines TGFβ1 (F) and IL10 (G) across groups. (H-K) Bar graphs showing the normalized expression (log2 scale) of pro-inflammatory genes IL6 (H), TNFSF9 (J), and MUC5B (K), and regulatory gene SOCS3 (I) across experimental groups before and after virus/allergen exposure. Data are represented as mean ± SD; ns, not significant; *P ≤ 0.05.

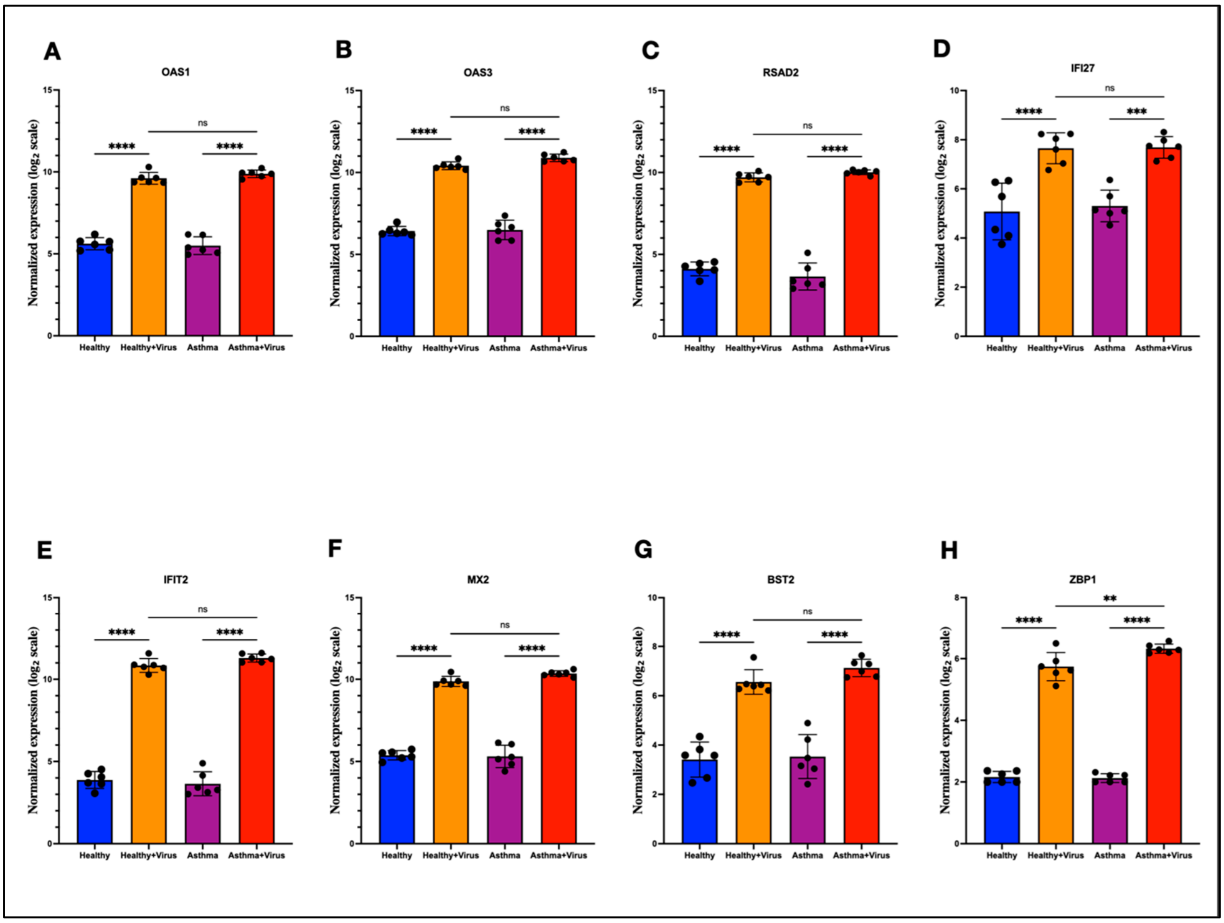

Figure S4. ISGs induction in airway epithelial cells from healthy and asthmatic subjects following rhinovirus infection. (A-H) Bar graphs showing the normalized expression (log_2 scale) of interferon-stimulated genes OAS1 (A), OAS3 (B), RSAD2 (C), IFI27 (D), IFIT2 (E), MX2 (F), BST2 (G), and ZBP1 (H) across the four experimental groups (Healthy, Health+virus, Asthma, and Asthma+virus). Data are represented as mean ± SD; ns, not significant; *P ≤ 0.05.

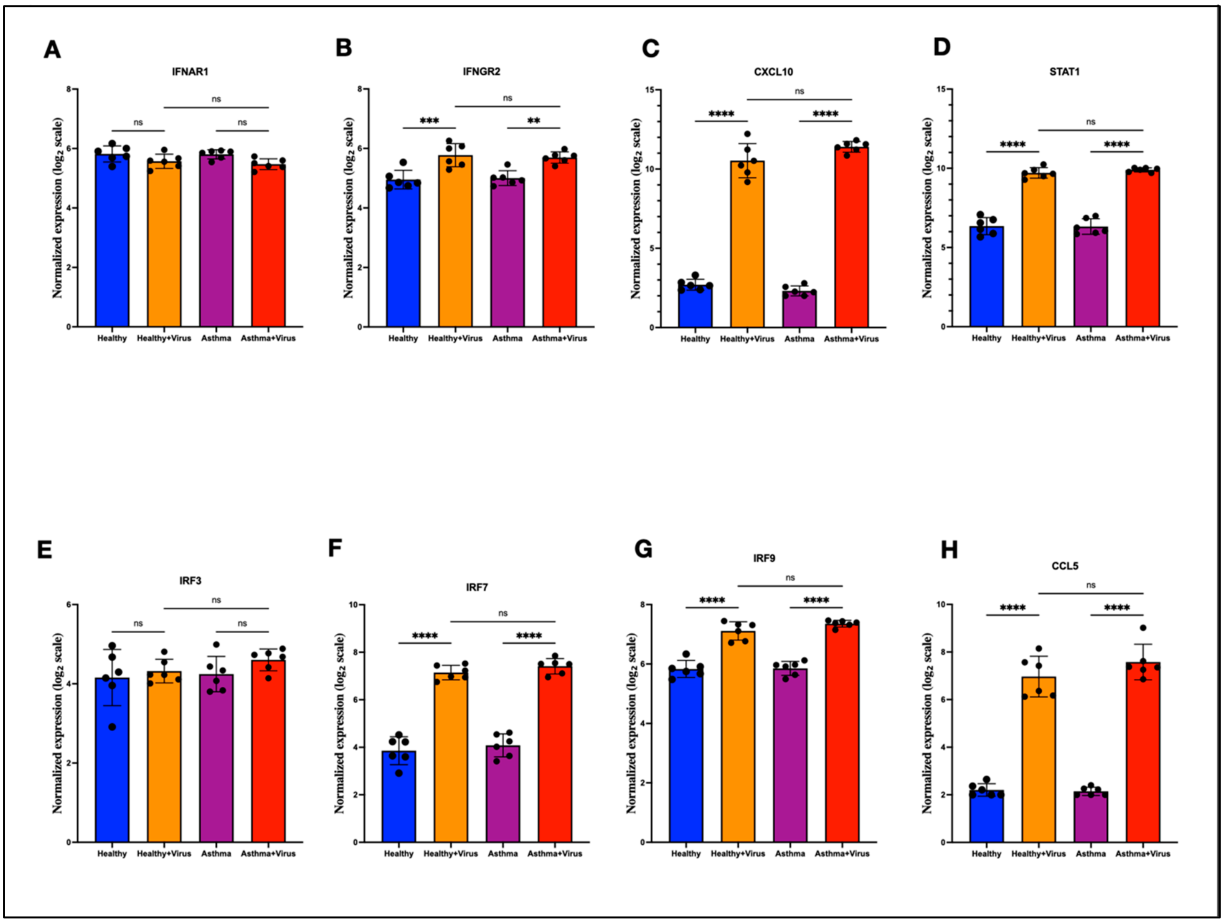

Figure S5. Induction of interferon pathway-related genes in airway epithelial cells from healthy and asthmatic donors following rhinovirus infection. (A-H) Bar graphs showing the normalized expression (log_2 scale) of interferon-related genes IFNAR1 (A), IFNGR2 (B), CXCL10 (C), STAT1 (D), IRF3 (E), IRF7 (F), IRF9 (G), and CCL5 (H) across the four experimental groups (Healthy, Health+virus, Asthma, and Asthma+virus). Data are represented as mean ± SD; ns, not significant; $^*P \leq 0.05$.

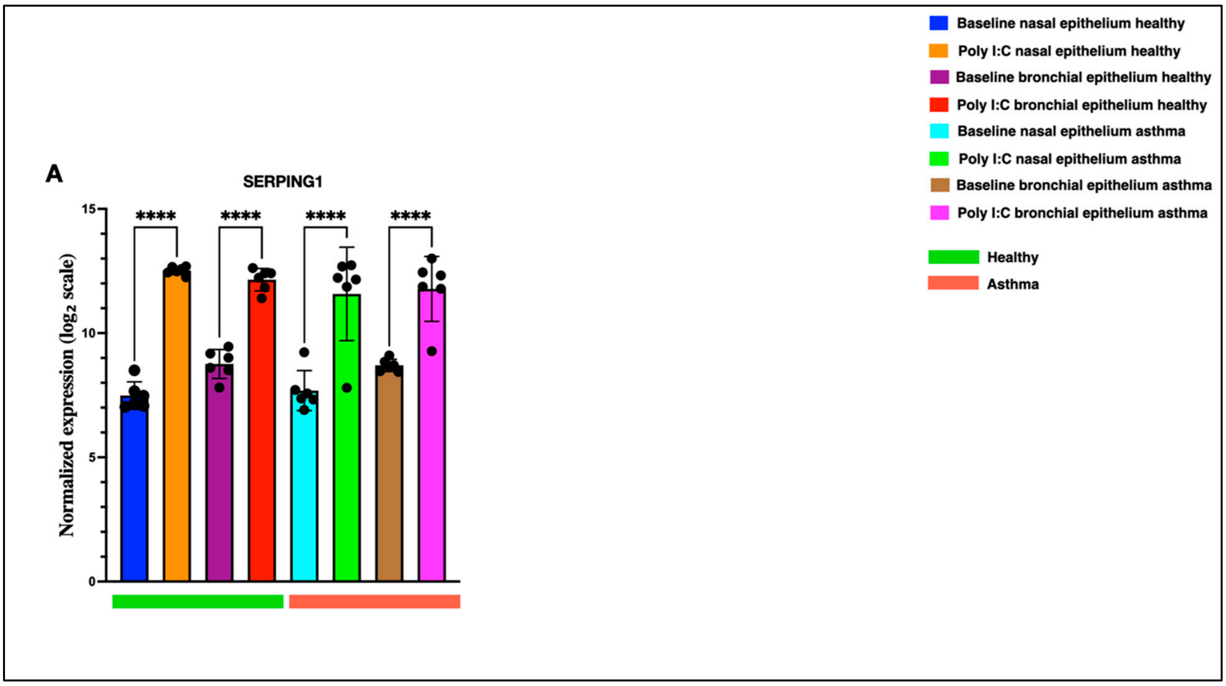

Figure S6. SERPING1 induction in nasal and bronchial epithelial cells after Poly I:C stimulation. (A) Bar graph showing the normalized expression (log2 scale) of SERPING1 gene in nasal and bronchial epithelial cells at baseline and after Poly I:C stimulation. Data are represented as mean ± SD; ns, not significant; *P ≤ 0.05.

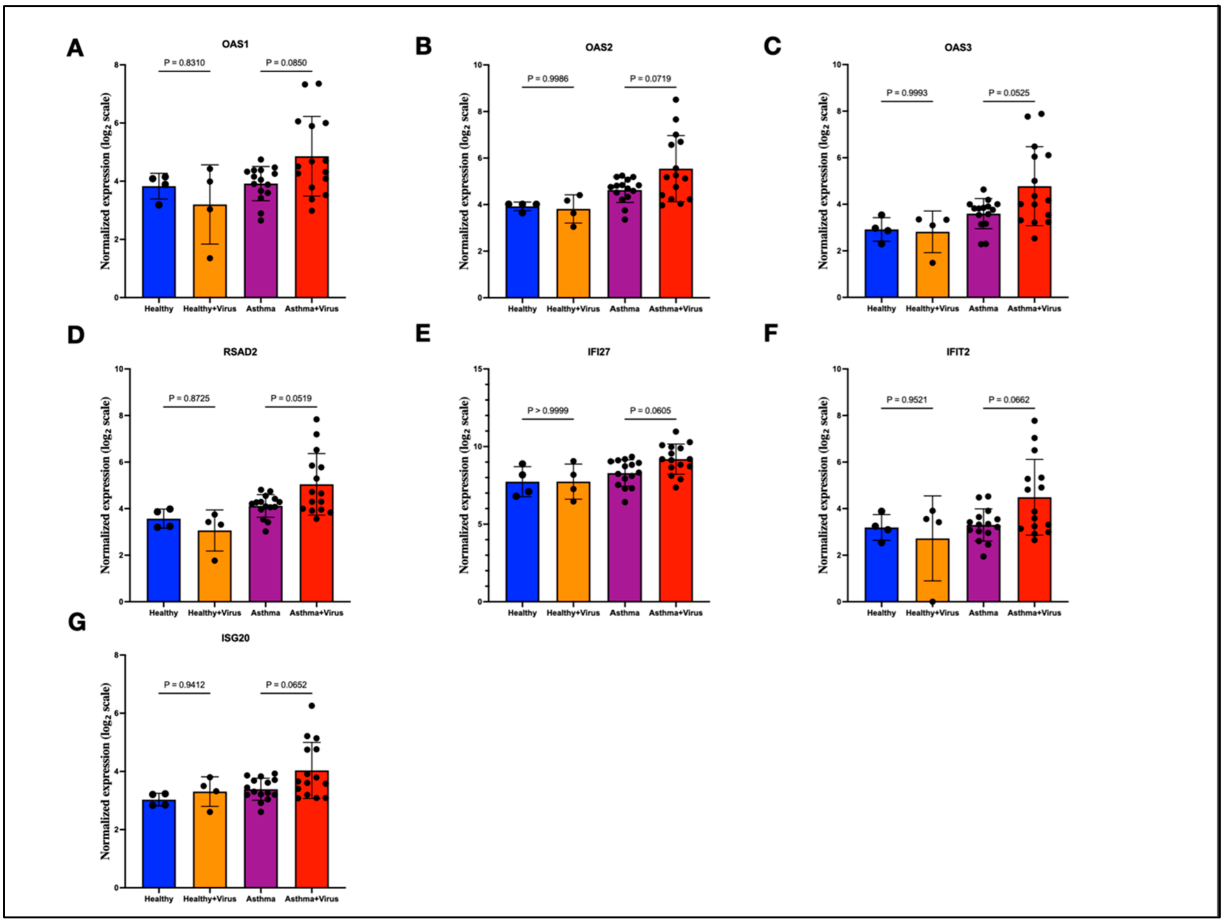

A
OAS1
P = 0.8310
P = 0.0850
B
OAS2
P = 0.9986
P = 0.0719
C
OAS3
P = 0.9993
P = 0.0525
D
RSAD2
P = 0.8725
P = 0.0519
E
IFI27
P > 0.9999
P = 0.0605
F
IFIT2
P = 0.9521
P = 0.0662
G
ISG20
P = 0.9412
P = 0.0652
Normalized expression (log2 scale)
Healthy
Healthy+Virus
Asthma
Asthma+Virus

Figure S7. Expression of ISGs in primary bronchial epithelial cells after RV16 challenge. Bronchial brushes were obtained from healthy and mild asthma donors before and after in-vivo RV16 infection. (A-G) Bar graphs showing the normalized expression (log_2 scale) of interferon-stimulated genes OAS1 (A), OAS2 (B), OAS3 (C), RSAD2 (D), IFI27 (E), IFIT2 (F), and ISG20 (G), across the four experimental groups (Healthy, Health+virus, Asthma, and Asthma+virus). Data are represented as mean ± SD; ns, not significant; *P ≤ 0.05.

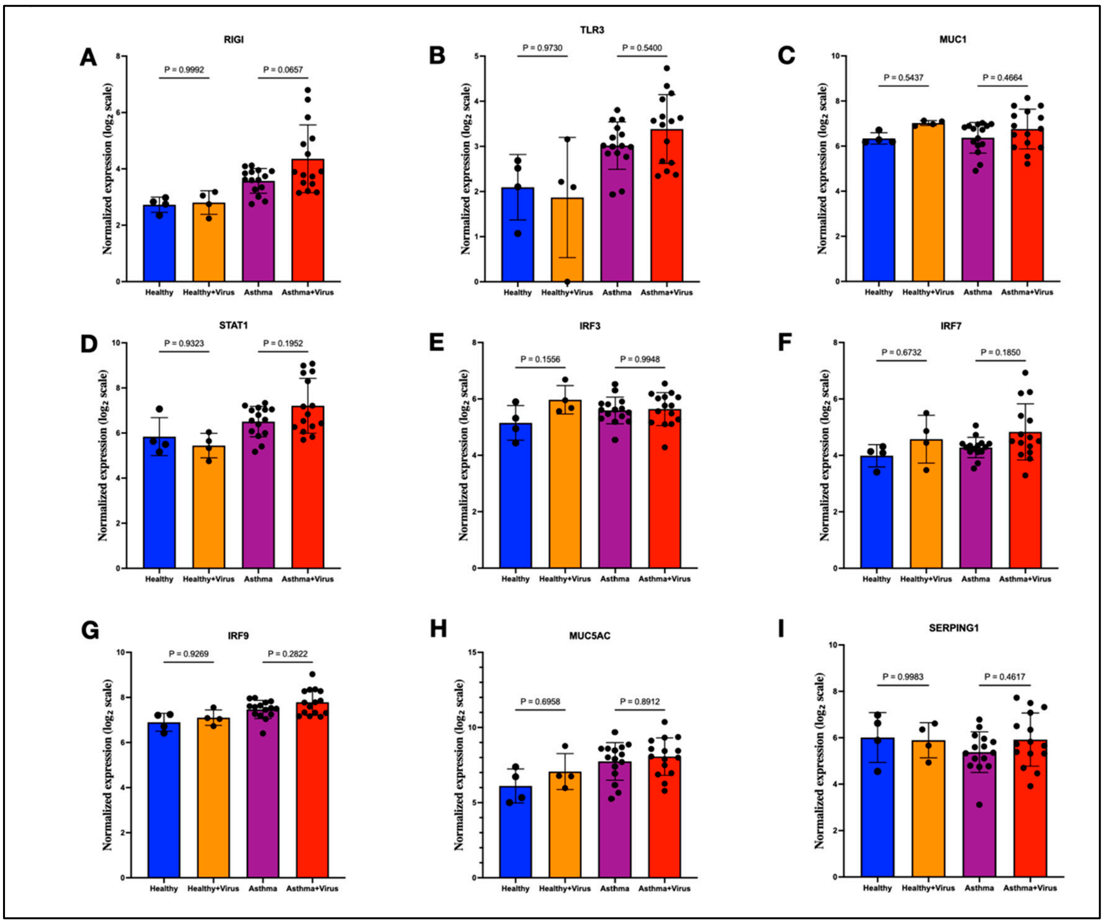

Figure S8. Expression of antiviral and interferon related-genes in primary bronchial epithelial cells after RV16 challenge. Bronchial brushes were obtained from healthy and mild asthma donors before and after in-vivo RV16 infection. (A-B) Bar graphs showing the normalized expression (log_2 scale) of viral dsRNA sensors RIG-I (A) and TLR3 (B) across the four experimental groups (Healthy, Health+virus, Asthma, and Asthma+virus). Bar graphs showing the normalized expression (log_2 scale) of epithelial/mucus-associated genes: MUC1 (C) and MUC5AC (H). (D–G) Interferon-associated transcription factors: STAT1 (D), IRF3 (E), IRF7 (F), and IRF9 (G). (I) Bar graph showing the normalized expression (log_2 scale) of complement regulator SERPING1 gene. Data are represented as mean ± SD; ns, not significant; *P ≤ 0.05.

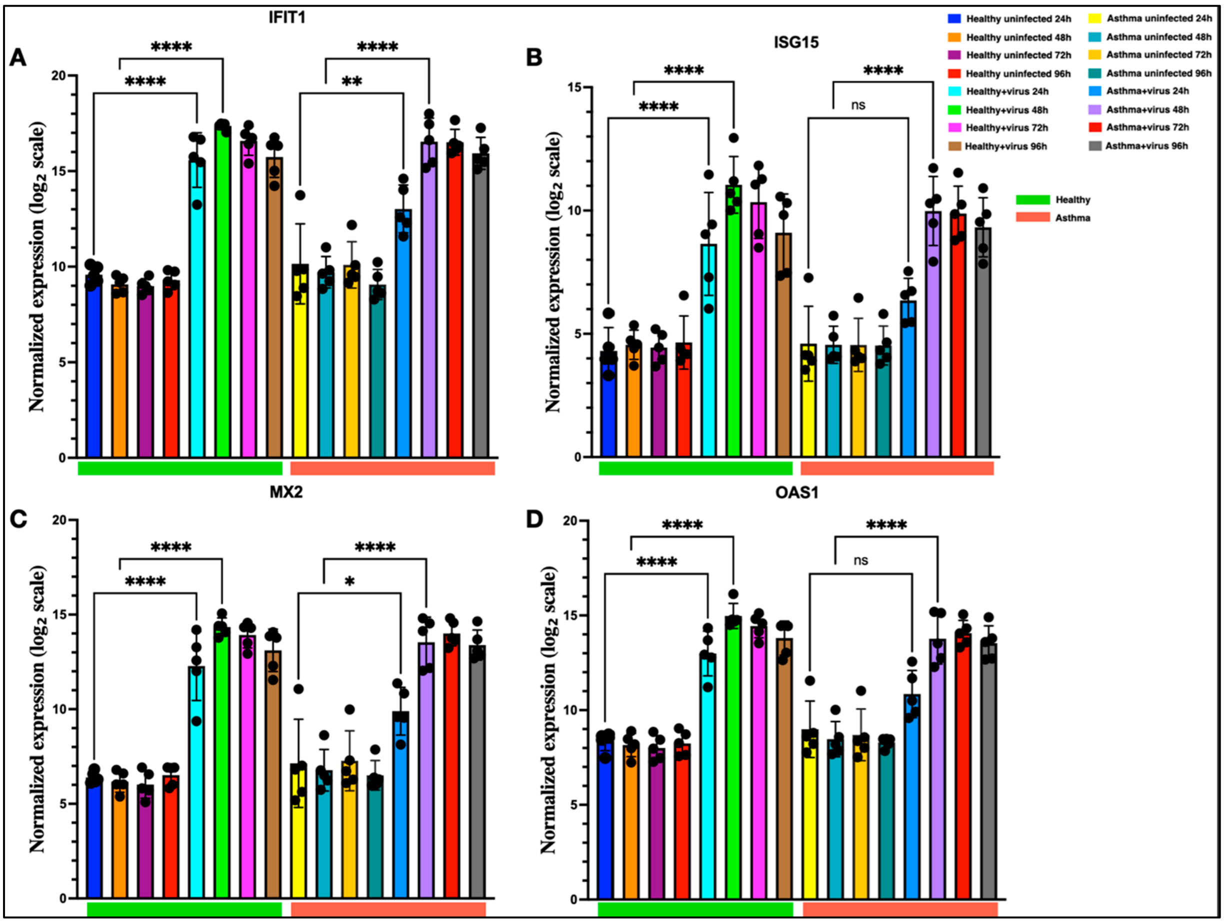

Figure S9. Induction of ISGs in healthy and asthmatic primary epithelial cells after rhinovirus 1A infection. (A–D) Bar graphs showing normalized expression (log_2 scale) of the interferon-stimulated genes IFIT1 (A), ISG15 (B), MX2 (C), and OAS1 (D) across groups (Healthy uninfected 24-96h, Healthy+virus 24-96h, Asthma uninfected 24-96h, and Asthma+virus 24-96h). Data are represented as mean ± SD; ns, not significant; *P ≤ 0.05.

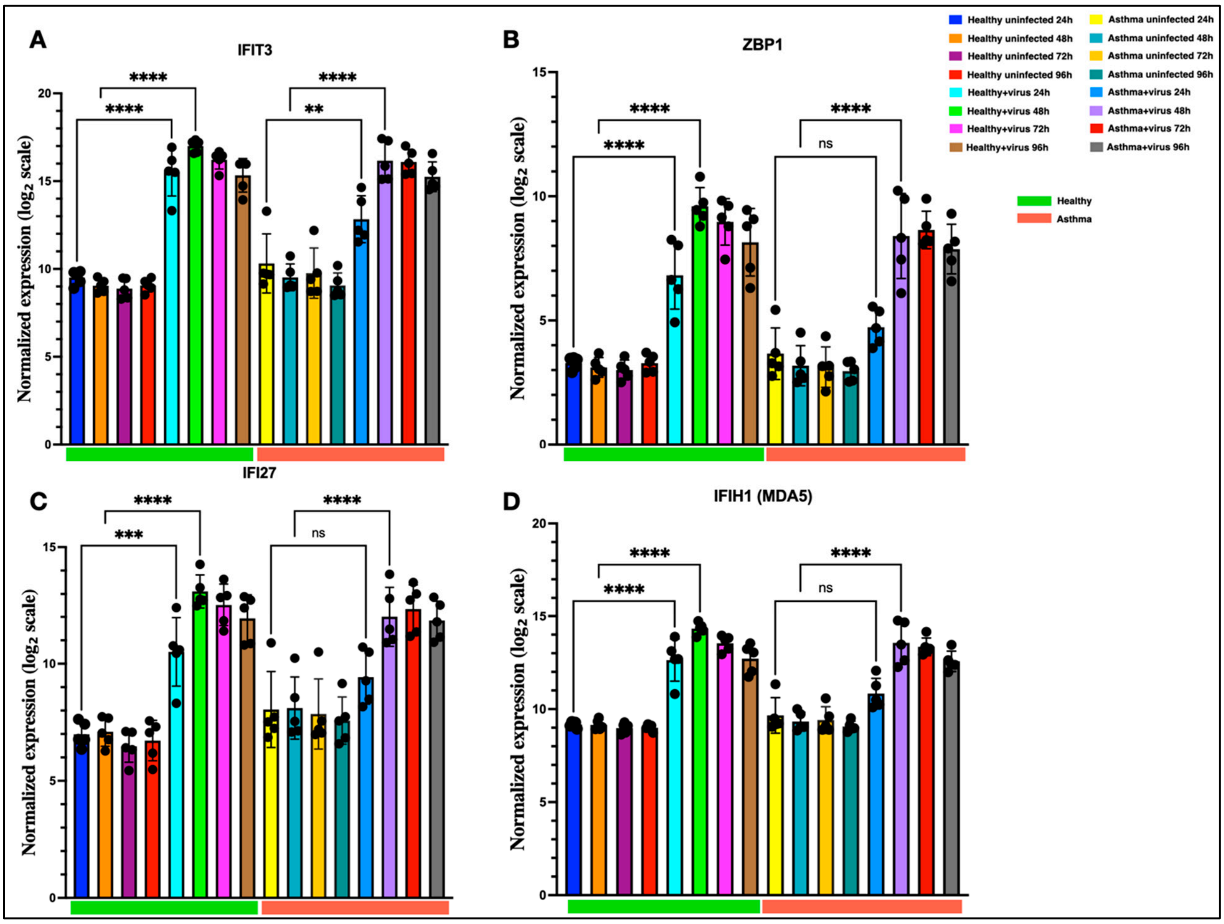

Figure S10. Induction of ISGs in healthy and asthmatic primary epithelial cells after Rhinovirus 1A infection. (A–D) Bar graphs showing normalized expression (log_2 scale) of the interferon-stimulated genes IFIT3 (A), ZBP1 (B), IFI27 (C), and IFIH1 (D) across groups (Healthy uninfected 24-96h, Healthy+virus 24-96h, Asthma uninfected 24-96h, and Asthma+virus 24-96h). Data are represented as mean ± SD; ns, not significant; *P ≤ 0.05.

Supplementary Table S1. Glossary of Major Interferon-Related, Inflammatory, and Epithelial Function-Associated Genes

Gene	Full Name	Category	Function
Oas1h	2'-5'-Oligoadenylate Synthetase 1H	Interferon-stimulated gene (ISG)	Contributes to interferon-induced antiviral immune responses.
Oasl2	2'-5'-Oligoadenylate Synthetase-Like 2		
OAS1-3	2'-5'-Oligoadenylate Synthetase 1-3		
OASL	2'-5'-Oligoadenylate Synthetase-Like		
IFI27	Interferon Alpha-Inducible Protein 27		
IFI6	Interferon Alpha-Inducible Protein 6		
IFI44	Interferon-Induced Protein 44		
IFI44L	Interferon-Induced Protein 44-Like		
MX1-2	MX Dynamin-Like GTPase 1-2		
IFIT1-3	Interferon-Induced Protein with Tetratricopeptide Repeats 1-3		
RSAD2 (Viperin)	Radical S-Adenosyl Methionine Domain Containing 2		

ISG15	Interferon-Stimulated Gene 15		
ISG20	Interferon-Stimulated Exonuclease Gene 20		
ZBP1	Z-DNA Binding Protein 1	Viral nucleic acid sensor / ISG	Senses viral nucleic acids and initiates antiviral immune responses.
DDX60	DExD/H-Box Helicase 60	Viral RNA sensor / ISG	Enhances RIG-I-like receptor signaling and promotes antiviral responses.
IL10	Interleukin-10	Anti-inflammatory cytokine	Suppresses excessive inflammation and maintains immune homeostasis during infection.
IL1B	Interleukin-1 Beta	Pro-inflammatory cytokine	Induces inflammation, immune cell recruitment and cytokine production.
IL6	Interleukin-6	Pro-inflammatory cytokine	Induces inflammation and contributes to antiviral immune responses and tissue repair.
MUC5AC	Mucin 5AC	Epithelial barrier/mucus gene	One of the main gel-forming mucin involved in mucus production and airway defense; usually increased in asthma.
MUC5B	Mucin 5B	Epithelial barrier/mucus gene	Gel-forming mucin important for mucociliary clearance.
TMPRSS2	Transmembrane Serine Protease 2	Epithelial protease / viral entry factor	Cleaves viral surface proteins to facilitate entry of certain viruses into host cells.
IFNAR1-2	Interferon Alpha and Beta Receptor Subunit 1-2	Type I interferon receptor	Mediate Type I interferon signaling and induction of ISGs.
IFNB1	Interferon Beta 1	Type I interferon	Induces antiviral gene expression and contributes to antiviral immune responses.

IFNL1-3	Interferon Lambda 1-3	Type III interferon	Mediates antiviral immunity at epithelial barriers.
SOCS3	Suppressor of Cytokine Signaling 3	Cytokine signaling regulator	Negatively regulates JAK/STAT signaling to prevent exaggerated interferon immune responses.
STAT1	Signal Transducer and Activator of Transcription 1	Interferon signaling molecule	Transcription factor that mediates interferon-induced expression of antiviral genes.
JAK/STAT	Janus Kinase/Signal Transducer and Activator of Transcription Pathway	Signaling pathway	Signaling pathway through which interferons and cytokines induce antiviral and inflammatory gene expression.
TNFSF9	Tumor Necrosis Factor Superfamily Member 9 (4-1BB Ligand)	Immune regulatory cytokine	Enhance T-cell activation and antiviral defense.
TGFB1	Transforming Growth Factor Beta 1	Immunoregulatory and remodeling cytokine	Induce anti-inflammatory responses, tissue repair and airway remodeling.
TGFBI	Transforming Growth Factor Beta-Induced Protein	Extracellular matrix protein	Enhances cell adhesion and tissue remodeling.
IFIH1 (MDA5)	Interferon-Induced Helicase C Domain-Containing Protein 1	Pattern recognition receptor	Detects viral double-stranded RNA and induces type I and III interferon production.
TLR3	Toll-Like Receptor 3	Pattern recognition receptor	Recognizes viral double-stranded RNA and induces innate antiviral immune responses.

RIG-I (DDX58)	DExD/H-Box Helicase 58	Pattern recognition receptor	Senses viral RNA and induces interferon-mediated antiviral responses.
cGAS (MB21D1)	Cyclic GMP-AMP Synthase	Cytosolic nucleic acid sensor	Detects cytosolic DNA and activates the STING pathway to induce interferon production.
IRF3	Interferon Regulatory Factor 3	Transcription factor	Transcription factor that induces production of type I and type III interferon after viral infection.
IRF7	Interferon Regulatory Factor 7	Transcription factor	Master regulator of type I interferon production and amplification of antiviral immune responses.
IRF9	Interferon Regulatory Factor 9	Transcription factor	Play a role in antiviral immune responses by contributing to type I interferons signaling.
CXCL10	C-X-C Motif Chemokine Ligand 10	Chemokine	Recruits activated T cells and natural killer cells to sites of viral infection.
CCL5	C-C Motif Chemokine Ligand 5 (RANTES)	Chemokine	Promotes antiviral immune responses by recruitments of leukocytes to sites of infection.
